# Extraction of Phenolics and Flavonoids from Four *Hosta* Species Using Reflux and Ultrasound-Assisted Methods with Antioxidant and *α*-Glucosidase Inhibitory Activities

**DOI:** 10.1155/2020/6124153

**Published:** 2020-12-12

**Authors:** Junwei He, Liangfa Wu, Li Yang, Boyuan Zhao, Chunlong Li

**Affiliations:** ^1^Jiangxi University of Traditional Chinese Medicine, Nanchang 330004, China; ^2^Jiangxi Provincial Institute for Drug Control, Nanchang 330029, China

## Abstract

The total phenolic and flavonoid contents (TPC and TFC) from the genus *Hosta* with antioxidant and *α*-glucosidase inhibitory activities were reported for the first time. Sixteen extracts from the aboveground and underground parts of the four *Hosta* species, including *H. plantaginea*, *H. ventricosa*, *H. ensata*, and *H. albofarinosa*, using reflux extraction (RE) and ultrasound-assisted extraction (UAE) techniques have high TPC and TFC with good antioxidant and *α*-glucosidase inhibitory activities. Furthermore, no significant differences on extraction yields, TPC, and TFC were found between RE and UAE techniques. Additionally, extracts from the aboveground parts of the four *Hosta* species had higher TPC, TFC, antioxidant, and *α*-glucosidase inhibitory activities compared to the underground parts by means of RE or UAE techniques. Lastly, the extracts of *H. albo-marginata* displayed a very remarkable *α*-glucosidase inhibitory activity compared to the positive control acarbose. The relationships of sixteen extracts of the four *Hosta* species were analyzed by RE and UAE techniques between extraction yields, TPC, TFC, antioxidant activity, and *α*-glucosidase inhibitory activity. The present study demonstrated that *H. plantaginea*, *H. ventricosa*, *H. ensata*, and *H. albofarinosa* could be new sources of natural antioxidants and antidiabetes for pharmaceutical and industrial purposes.

## 1. Introduction

*Hosta* Tratt. is a genus belonging to the family Liliaceae, which comprises about 50 species distributed mainly in the temperate and subtropical zones of East Asia and Russia, but most species have been found in China, Japan, and Korea, and most of them are commonly used for ornamentally purpose or traditional folk medicines [1, 2]. Only four native species have been found in China, namely, *H. plantaginea* (Lam.) Aschers, *H. ventricosa* (Salisb.) Stearn, *H. ensata* F. Maekawa, and *H. albofarinosa* D.Q.Wang, which are widely cultivated in parks and/or commonly used as traditional Chinese medicines [1]. Moreover, *H. albo-marginata* (Hook.) Ohwi originated in Japan and is cultivated as an ornamental plant in many regions of China [1]. The genus *Hosta* is a particularly rich source of polyphenolics and flavonoids, which have been linked to antioxidant and anti-inflammatory effects [2–6]. However, the total phenolic and flavonoid contents (TPC and TFC), as well as the antioxidant and *α*-glucosidase inhibitory activities, have not been reported from the genus *Hosta*, except that some flavonoids were moderate against the DPPH free radical scavenging activity in our previous articles [3–6].

Polyphenolics and flavonoids are the important class of secondary metabolites and are widely distributed in plant kingdoms, which have attracted considerable interest due to their beneficial effects on human health and pharmaceutical and industrial research, showing a broad spectrum of biological activities, such as antioxidant, enzyme inhibition, anti-inflammatory properties, and others [7–9]. It is believed that sufficient intake of polyphenol-rich plant foods or extracts can reduce the risk of diabetes, hypertension, obesity, and cancers, which can be partly explained by the strong antioxidant and enzyme inhibitory activities of polyphenolics [8]. Furthermore, the bioactivity of polyphenolics is strongly dependent on the parts of the plant and extraction techniques [10, 11]. For these reasons, it is very important to select the appropriate combination of these variables to improve the extraction and functionality of polyphenolics. In addition, reflux extraction (RE) and ultrasound-assisted extraction (UAE) are the commonly followed methods used in plant materials [12–14].

Therefore, the present work is an attempt to compare the effectiveness of RE and UAE technologies in the extraction of total phenolics and flavonoids from different varieties of the four *Hosta* species, including *H. plantaginea*, *H. ventricosa*, *H. ensata*, and *H. albofarinosa*, as well as associating the TPC and TFC with antioxidant and *α*-glucosidase inhibitory activities, to better contribute to the promotion of the use of natural ingredients as an important and safe alternative to antioxidant and *α*-glucosidase inhibitory products. To the best of our knowledge, this is the first report on the TPC and TFC with antioxidant and *α*-glucosidase inhibitory activities of the genus *Hosta*.

## 2. Materials and Methods

### 2.1. Chemicals and Reagents

Folin-Ciocalteau reagent, 1,1-diphenyl-2-picrylhydrazyl (DPPH), 2,2′-azino-bis(3-ethylbenzothiazoline-6-sulphonic acid) (ABTS), potassium persulfate (K_2_S_2_O_8_), dimethylsulfoxide (DMSO), and *α*-glucosidase were purchased from Sigma-Aldrich (St. Louis, MO, USA). Gallic acid, rutin, acarbose, vitamin c (Vc), Na_2_CO_3_, NaNO_2_, Al(NO_3_)_3_, NaOH, and p-Nitrophenyl-*α*-D-glucopyranoside (pNPG) were purchased from Aladdin (Shanghai, China). Phosphate-buffered saline (PBS, pH 6.8 and 7.4) was purchased from Nanjing SenBeiJia Biological Technology Co., Ltd. (Nanjing, China).

### 2.2. Plant Materials

Fresh whole plant of *H. ventricosa* was collected from the experimental farm of the Jiangxi University of Traditional Chinese Medicine, Nanchang, China, before the flowering stage (June 2016). At the same time, the fresh whole plant of *H. plantaginea*, *H. ensata*, and *H. albofarinosa* was collected from the Beizhangling farm, Mingguandian Township, Anguo City, Hebei Province, China. These plants were identified by Prof. Guoyue Zhong at Jiangxi University of Traditional Chinese Medicine.

### 2.3. Extraction Procedure

One gram of sample was used in 30 mL of 70% ethanol. The extracts obtained by either RE or UAE techniques were filtered and evaporated to dryness using a rotary evaporator, and the residues were stored at 4°C until use. The resulting extracts were coded as a combination of the type of part used and the development approaches for each extraction technique. The extraction yields and abbreviations of these extracts are given in Table 1.

#### 2.3.1. Reflux Extraction (RE)

For the RE, a dry sample of each plant material (1.0 g) was added to a round-bottom flask and thoroughly soaked in 30 mL of 70% ethanol at room temperature for 12 h, after which it was heated until the solvent reached boiling point and was heated under reflux at 90°C for 90 min.

#### 2.3.2. Ultrasound-Assisted Extraction (UAE)

For the UAE, a dry sample of each plant material (1.0 g) was thoroughly soaked in 30 mL of 70% ethanol at room temperature for 12 h, after which it was sonicated in an ultrasonic bath (the maximum power of 200 W at a frequency of 40 KHz, Kunshan, China) at 60°C for 60 min.

### 2.4. Total Phenolic and Flavonoid Contents (TPC and TFC)

The TPC was determined using the Folin-Ciocalteau reagent according to the method of Sun et al. (2017) with modifications using gallic acid as the standard [15]. Briefly, 0.1 mL of a fresh extract solution was mixed with 0.5 mL of Folin-Ciocalteau reagent for 5 min at room temperature. Then, 2.0 mL of 7.5% (w/v) Na_2_CO_3_ was added, and the solution was vortexed and adjusted to 2.4 mL with distilled water. After 90 min, *A*_750_ was measured in a UV-1800 spectrophotometer (Shimadzu, Kyoto, Japan) using distilled water as a blank. The TPC was calculated based on the standard curve of gallic acid, and the results are expressed as milligrams of gallic acid equivalent per gram of extract (mg GAE/g extract). All tests were performed in triplicate, and the values obtained from the experiments were averaged.

The TFC was determined by the aluminium nitrate method according to Sun et al. (2017) with modifications using rutin as the standard [15]. Briefly, 0.25 mL of a fresh extract solution was mixed with 0.15 mL of 5% NaNO_2_ (w/v) for 5 min at room temperature. Then, 0.15 mL of 10% Al(NO_3_)_3_ was added and mixed. After another 5 min, 2.0 mL of 1 M NaOH solution was added and mixed before the volume was adjusted to 5 mL with distilled water. *A*_510_ was measured after 10 min at room temperature in a UV-1800 spectrophotometer (Shimadzu, Kyoto, Japan) using distilled water as a blank, and the results are expressed in mg rutin equivalent per gram of extract (mg RE/g extract) in accordance with the calibration curve constructed using rutin as the standard solution. All tests were performed in triplicate, and the values obtained from the experiments were averaged.

### 2.5. Antioxidant Assay

#### 2.5.1. DPPH Free Radical Scavenging Activity

The DPPH radical scavenging activity of the tested samples was provided in our previously published articles [3]. In a 96-well microplate, 150 *μ*L of DPPH solution (200 *μ*M) was added to 50 *μ*L of the test sample in methanol at different concentrations. The mixture was stirred and allowed to stand for 30 min at 30°C. The absorbance of the resulting solution was determined at 517 nm using a Multiskan Go (Thermo Fisher Scientific, Inc., Waltham, MA, USA). The DPPH radical scavenging activity was calculated by the following equation: DPPH scavenging activity (%) = [1 − (*A*_sample_–*A*_blank_)/*A*_control_] × 100, where *A*_sample_ represents the absorbance of the sample and DPPH, *A*_blank_ represents the absorbance of the sample and CH_3_OH, and *A*_control_ represents the absorbance of DPPH and CH_3_OH. The IC_50_ value is calculated as the concentration required to scavenge *50%* of the *DPPH* free *radicals* and was obtained by plotting the DPPH scavenging percentage of each sample against the sample concentration. Vc was used as a positive control in this experiment. All tests were performed in triplicate, and the values obtained from the experiments were averaged.

#### 2.5.2. ABTS Free Radical Scavenging Activity

The ABTS free radical scavenging activity of the tested samples was carried out using the method reported by Sun et al. (2017) with minor modifications [15]. The ABTS stock solution was prepared by adding 88 *μ*L of K_2_S_2_O_8_ (140 mM) and 5 mL of ABTS salt (7 mM) in a brown bottle. The stock solution was stored in the dark for 12 h at room temperature before use. The ABTS^+^ radical solution was diluted with PBS (pH 7.4) until an absorbance value of 0.70 ± 0.02 was reached at 734 nm. In a 96-well microplate, 195 *μ*L of the diluted ABTS^+^ radical solution was mixed with 10 *μ*L of various concentrations of the test samples. The mixture was allowed to react for 106 min, and the absorbance at 734 nm was measured using a Multiskan Go (Thermo Fisher Scientific, Inc., Waltham, MA, USA). The ABTS^+^ radical scavenging activity was calculated by the following equation: ABTS scavenging activity (%) = [1 − (*A*_sample_–*A*_blank_)/*A*_control_] × 100, where *A*_sample_ represents the absorbance of the sample and ABTS, *A*_blank_ represents the absorbance of the sample and CH_3_OH, and *A*_control_ represents the absorbance of ABTS and CH_3_OH. The IC_50_ value is calculated as the concentration required to scavenge *50%* of the ABTS free *radicals* and was obtained by plotting the ABTS scavenging percentage of each sample against the sample concentration. Vc was used as a positive control in this experiment. All tests were performed in triplicate, and the values obtained from the experiments were averaged.

### 2.6. *α*-Glucosidase Inhibitory Assay

The inhibitory activity of *α*-glucosidase (E.C. 3.2.1.20, from the yeast Saccharomyces cerevisiae) was determined according to the modified method of a previously reported method [16]. Briefly, mixtures of 100 *μ*L of enzyme solution (1 unit/mL) and 50 *μ*L of the sample were incubated in a 96-well plate (Fisher Scientific, USA) at 25°C for 10 min, followed by the addition of 50 *μ*L of pNPG 2.5 mM to each well, and incubation at 25°C for 5 min. At the end of the reaction, the absorbance was determined at 405 nm using a Multiskan Go (Thermo Fisher Scientific, Inc., Waltham, MA, USA). The enzyme, the sample, and the pNPG were dissolved in PBS (0.1 M, pH 6.8). The assay was performed in triplicate with six different concentrations, and acarbose was used as a positive control. The *α*-glucosidase inhibition percentage was calculated by the equation: inhibition (%) = [1 − (*A*_sample_–*A*_blank_)/*A*_control_] × 100, where *A*_sample_ represents the absorbance of the enzyme+sample + pNPG, *A*_blank_ represents the absorbance of the sample+PBS + pNPG, and *A*_control_ represents the absorbance of the enzyme+PBS+pNPG. The IC_50_ value (mg/mL) is defined as the concentration that inhibits 50% of the *α*-glucosidase activity.

### 2.7. Statistical Analysis

Graphpad Prism6 was used for statistical analysis, and the data were presented as the means ± standard deviation (SD). One-way analysis of variance (ANOVA) and Tukey's test were used for comparison differences groups. Differences with *P* < 0.05 indicated statistical significance.

## 3. Results and Discussion

### 3.1. Extraction Yield

Sixteen extracts of four *Hosta* species (*H. plantaginea*, *H. ventricosa*, *H. ensata*, and *H. albofarinosa*) were obtained using different plant parts (aboveground part and underground part) and extraction techniques (RE and UAE), the extraction yields as shown in Table 1.

In terms of the extraction yields, no significant differences were found between RE and UAE techniques. Meanwhile, extracts from the underground parts of four *Hosta* species had higher extraction yields compared to the aboveground parts (*P* < 0.01) by RE or UAE techniques. On the other hand, *H. albo-marginata* had the highest extraction yields from the underground part by both extraction techniques. Furthermore, *H. ensata* extracts displayed the significantly lowest extraction yields for different parts and both extraction techniques.

### 3.2. TPC Determination

**Figure 1:**
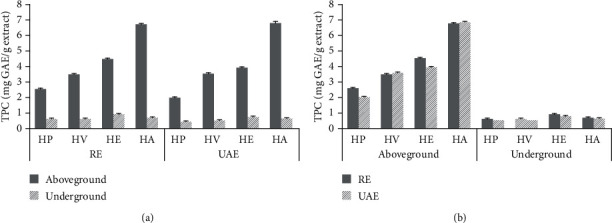
Comparison of the TPC from four *Hosta* species corresponding to different parts (a) and extraction techniques (b).

In terms of the TPC, no significant differences were found between the RE and UAE techniques (Figure 1). On the other hand, the amount of TPC extracts in RE or UAE ranged from 2.02 ± 0.01 to 6.83 ± 0.07 and from 0.50 ± 0.01 to 0.94 ± 0.03 mg GAE/g extract in the aboveground parts and underground parts of four *Hosta* species, respectively. In the case of RE or UAE extracts, the TPC for these extracts has the order of HA > HE > HV > HP for the aboveground parts and the order of HE > HA > HV ≈ HP for the underground parts.

### 3.3. TFC Determination

**Figure 2:**
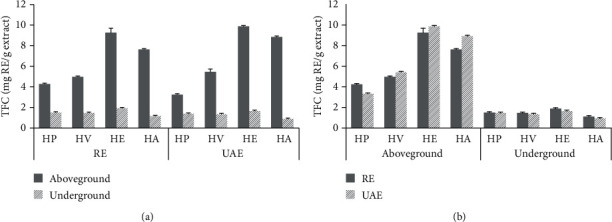
Comparison of the TFC from four *Hosta* species corresponding to different parts (a) and extraction techniques (b).

As shown in Figure 2, there are no significant differences of the TFC values for the sixteen extracts of four *Hosta* species by RE or UAE techniques. Moreover, the amount of TFC in RE or UAE extracts ranged from 3.36 ± 0.05 to 9.87 ± 0.10 and from 0.93 ± 0.07 to 1.93 ± 0.15 mg RE/g extract in the aboveground parts and underground parts of four *Hosta* species, respectively. In the case of RE or UAE extracts, the TFC for these extracts has the order of HE > HA > HV > HP for the aboveground parts, while the order of HE > HP > HV > HA for the underground parts.

The results of the extraction yield, TPC, and TFC in our experiments established that the UAE method can be a viable alternative to the RE method, which usually involves many disadvantages, such as long extraction time, large amounts of solvent, and high temperatures [12–14]. However, further investigations are required to obtain the optimization parameters of the extraction process in pharmaceutical and industrial research.

### 3.4. Antioxidant Activity

**Figure 3:**
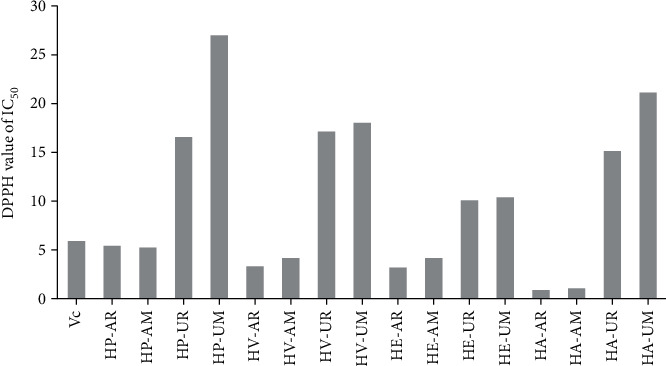
IC_50_ values for the DPPH free radical scavenging activities of Vc (*μ*g/mL) and four *Hosta* species extracts (mg/mL).

**Figure 4:**
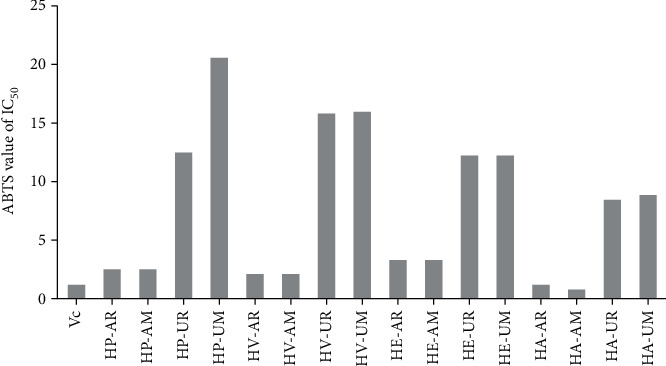
IC_50_ values for the ABTS free radical scavenging activities of Vc (*μ*g/mL) and four *Hosta* species extracts (mg/mL).

The DPPH and ABTS free radical scavenging activity assays are mostly used to evaluate the antioxidant activity of crude extracts and/or pure compounds. Hence, the antioxidant activity of the sixteen extracts of the four *Hosta* species was evaluated using ABTS and DPPH assays, and the results are shown in Figures 3 and 4. For the extracts of RE or UAE, the aboveground parts for both DPPH and ABTS assays displayed significantly higher antioxidants than that of the underground parts from the four *Hosta* species (*P* < 0.05). In the case of the RE or UAE extracts, it was found that the ability of the extracts to scavenge the DPPH radical is of the order of HA > HE ≈ HV > HP for the aboveground parts. For extracts from underground parts, the DPPH radical scavenging activity for these extracts is of the order of HE > HV > HA > HP and HE > HA > HV > HP for RE and UAE extraction techniques, respectively (Figure 3).

In the case of RE or UAE extracts, the ABTS radical scavenging activity for these extracts is of the order of HA > HV > HP > HE for the aboveground parts and the order of HA > HE > HP > HV for the underground parts (Figure 4).

In this experiment, the DPPH method yielded higher IC_50_ values than those found in the ABTS assay and, consequently, lower antioxidant activity of the four *Hosta* species, including *H. plantaginea*, *H. ventricosa*, *H. ensata*, and *H. albofarinosa*, except for the three extracts of HE-AR, HE-UR, and HE-UM. Furthermore, our results showed these differences in the IC_50_ values of DPPH and ABTS for the same extract in all cases, which may due to differences mechanisms in both assays [10, 17]. It is worthy that the antioxidant effects closely related to the presence of multiple hydroxyl groups and their arrangement in the structures of phenolics and flavonoids [3, 8, 18].

### 3.5. *α*-Glucosidase Inhibition Activity

**Figure 5:**
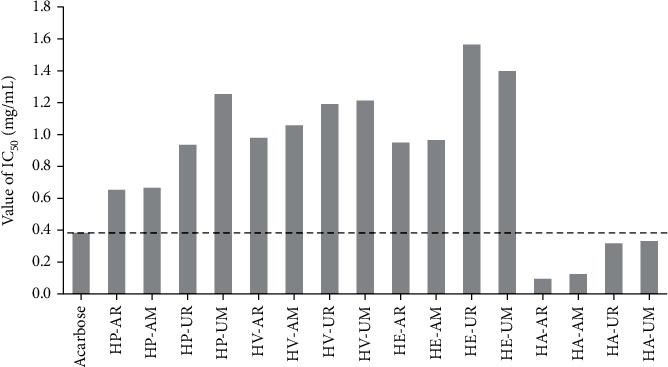
The *α*-glucosidase inhibitory activity of acarbose and four *Hosta* species extracts.

As shown in Figure 5, all of these extracts were found to exhibit strong or moderate *α*-glucosidase inhibitory activity. Moreover, the extracts of *H. albo-marginata* (HA) displayed a very remarkable *α*-glucosidase inhibitory activity with IC_50_ values ranging from 0.093 to 0.330 mg/mL compared to the positive control acarbose with an IC_50_ value of 0.378 mg/mL. In terms of the *α*-glucosidase inhibitory activity, some differences were found between the RE and UAE extraction techniques. On the other hand, the *α*-glucosidase inhibitory activity on RE or UAE extracts ranged from 0.093 to 0.983 and from 0.313 to 1.568 mg/mL in the aboveground parts and underground parts of four *Hosta* species, respectively. In the case of RE or UAE extracts, the order is HA > HP > HE > HV for the aboveground parts. For the extracts of the underground parts, the *α*-glucosidase inhibitory activity for these extracts is of the order of HE > HA > HP > HV and HE > HV > HA > HP for RE and UAE extraction techniques, respectively.

Oxidative damage caused by free radicals is considered to be associated with many human diseases including diabetes, hypertension, obesity, and cancers [19, 20]. Apart from the antioxidant effect, phenolics and flavonoids may also play a key role in the inhibition of the *α*-glucosidase activity [20–24]. The results of our experiment seem to agree with this finding, indicating that phenolics and flavonoids are not only antioxidants but also contributors to the *α*-glucosidase inhibitory activity in the four *Hosta* species.

In addition, HA contained higher total flavonoids and total phenolics, but has the weakest *α*-glucosidase inhibitory activity compared to the other three species in this experiment. Based on these evidences, we suggested that other constituents, such as phenylpropanoids and triterpenoids, might be the potential *α*-glucosidase inhibitory activity constituents in the four *Hosta* species [23–26].

### 3.6. Pearson Correlation Analysis

To better appreciate the relationships between the extraction yield, TPC, TFC, antioxidant activity, and *α*-glucosidase inhibitory activity, Pearson correlation analysis under RE and UAE extractions of the four *Hosta* species, including *H. plantaginea*, *H. ventricosa*, *H. ensata*, and *H. albofarinosa*, was analyzed.

Under the “yield” parameter for RE extracts (Table 2), the correlations between yield and TPC/TFC were negative and highly significant (*P* < 0.01). This result suggests that not all of the extracted compounds correspond to phenolics and/or flavonoids. On the other hand, the correlations between yield and ABTS were positive and highly significant phenolic and flavonoid compounds. This result means that a higher yield promotes the ABTS free radical scavenging activity. Meanwhile, the pairs of yield–DPPH and yield–*α*-glucosidase inhibitory activity correlations were not significant. The same behavior was observed for UAE extracts. Thus, differences in extraction techniques are appropriate for obtaining high yield of phenolic and flavonoid compounds.

As for the “TPC”, it was negatively correlated with DPPH (*P* < 0.01) and ABTS (*P* < 0.05) for both RE and UAE extracts, while the “TFC” parameter was negatively correlated with DPPH (*P* < 0.01 or *P* < 0.05) and ABTS (*P* < 0.05) in both cases. Therefore, it can be concluded that the presence of different types of phenolics and/or flavonoids contributes to the DPPH and ABTS free radical scavenging activities of four *Hosta* species extracts. On the other hand, it is due to the difference of the number and position of hydroxyl groups in phenolics and/or flavonoids [3, 8, 18]. Finally, DPPH had a significant positive correlation with the *α*-glucosidase inhibitory activity (*P* < 0.05) in UAE extracts, whereas ABTS had a significant positive correlation with the *α*-glucosidase inhibitory activity (*P* < 0.01) for both RE and UAE extracts.

Hence, this information is useful in elucidating the relationships among the extraction yields, TPC, TFC, antioxidant activity, and *α*-glucosidase inhibitory activity of the four *Hosta* species, including *H. plantaginea*, *H. ventricosa*, *H. ensata*, and *H. albofarinosa* by RE and UAE techniques.

## 4. Conclusions

To summarize our findings, this is the first report on the total phenolic and flavonoid contents with antioxidant and *α*-glucosidase inhibitory activities of the genus *Hosta*. This work has shown that extracts from four *Hosta* species, including *H. plantaginea*, *H. ventricosa*, *H. ensata*, and *H. albofarinosa*, have high yields of TPC and TFC, as well as good antioxidant and *α*-glucosidase inhibitory activities. In addition, no significant differences on extraction yields, TPC, and TFC were found between RE and UAE techniques. On the other hand, the extracts of the aboveground parts of four *Hosta* species had higher TPC, TFC, antioxidant, and *α*-glucosidase inhibitory activities compared to the underground parts by means of RE or UAE techniques. In addition, the extracts of *H. albo-marginata* (HA) displayed a very remarkable *α*-glucosidase inhibitory activity compared to the positive control acarbose. However, the compounds responsible for the antioxidant and *α*-glucosidase inhibitory activities are currently unknown. Further, work on *H. plantaginea*, *H. ventricosa*, *H. ensata*, and *H. albofarinosa*, in isolating these compounds and explaining the mechanisms responsible for their antioxidant and *α*-glucosidase inhibitory activities, is warranted.

## Figures and Tables

**Table 1:** Extraction yields (EY) and abbreviations (Ab) of the four *Hosta* species.

Plants	Parts	RE		UAE	
		EY (%)	Ab	EY (%)	Ab
*H. plantaginea*	Aboveground	40.89 ± 0.33	HP-AR	40.18 ± 0.54	HP-AM
	Underground	51.61 ± 1.33	HP-UR	51.81 ± 0.47	HP-UM
*H. ventricosa*	Aboveground	36.10 ± 0.68	HV-AR	35.09 ± 0.30	HV-AM
	Underground	50.49 ± 1.56	HV-UR	53.21 ± 0.32	HV-UM
*H. ensata*	Aboveground	20.91 ± 0.46	HE-AR	19.79 ± 0.32	HE-AM
	Underground	44.73 ± 1.24	HE-UR	45.81 ± 0.15	HE-UM
*H. albo-marginata*	Aboveground	39.59 ± 0.04	HA-AR	39.98 ± 0.42	HA-AM
	Underground	60.78 ± 0.18	HA-UR	62.25 ± 0.04	HA-UM

Results are expressed as the mean ± SD (*n* = 3). RE: reflux extraction; UAE: ultrasound-assisted extraction; HP-AR: the aboveground part of *H. plantaginea* using reflux extraction; HP-AM: the aboveground part of *H. plantaginea* using ultrasound-assisted extraction; HP-UR: the underground part of *H. plantaginea* using reflux extraction; HP-UM: the underground part of *H. plantaginea* using ultrasound-assisted extraction; HV-AR: the aboveground part of *H. ventricosa* using reflux extraction; HV-AM: the aboveground part of *H. ventricosa* using ultrasound-assisted extraction; HV-UR: the underground part of *H. ventricosa* using reflux extraction; HV-UM: the underground part of *H. ventricosa* using ultrasound-assisted extraction; HE-AR: the aboveground part of *H. ensata* using reflux extraction; HE-AM: the aboveground part of *H. ensata* using ultrasound-assisted extraction; HE-UR: the underground part of *H. ensata* using reflux extraction; HE-UM: the underground part of *H. ensata* using ultrasound-assisted extraction; HA-AR: the aboveground part of *H. albo-marginata* using reflux extraction; HA-AM: the aboveground part of *H. albo-marginata* using ultrasound-assisted extraction; HA-UR: the underground part of *H. albo-marginata* using reflux extraction; HA-UM: the underground part of *H. albo-marginata* using ultrasound-assisted extraction.

**Table 2:** Pearson correlation coefficients between different assays^a^ under influence of extraction conditions (*n* = 3)^b^.

	RE extracts					UAE extracts				
	TPC	TFC	DPPH	ABTS	*α*-Glu	TPC	TFC	DPPH	ABTS	*α*-Glu
Yield	-0.690^∗∗^	-0.890^∗∗^	0.795^ns^	0.615^∗^	-0.133^ns^	-0.632^∗∗^	-0.854^∗∗^	0.769^ns^	0.617^ns^	-0.069^ns^
TPC		0.913^ns^	-0.898^∗∗^	-0.828^∗^	-0.513^ns^		0.907^ns^	-0.795^∗∗^	-0.776^∗^	-0.570^ns^
TFC			-0.876^∗^	-0.792^ns^	-0.311^ns^			-0.777^∗∗^	0.727^∗^	-0.362^ns^
DPPH				0.909^ns^	0.28^ns^				0.895^ns^	0.308^∗^
ABTS					0.561^∗∗^					0.605^∗∗^

^a^TPC: total phenolic contents; TFC: total flavonoid contents; DPPH: DPPH free radical scavenging activity; ABTS: ABTS free radical scavenging activity; *α*-glu: *α*-glucosidase inhibitory activity; ^b^RE extracts: reflux extracts; UAE extracts: ultrasound assisted extracts. ^ns^Not significant, ^∗^significant at *P* < 0.05, ^∗∗^significant at *P* < 0.01.

## Data Availability

The data used to support the findings of this study are available from the corresponding author upon request.
